# Theoretical and experimental studies of an oseltamivir–triazole-based thermoresponsive organogel†

**DOI:** 10.1039/c9ra02463h

**Published:** 2019-07-04

**Authors:** Sumit Kumar, Lidong Wu, Neha Sharma, Ayushee, Kumar Kaushik, Maria Grishina, Bhupendra S. Chhikara, Poonam, Vladimir Potemkin, Brijesh Rathi

**Affiliations:** Laboratory for Translational Chemistry and Drug Discovery, Department of Chemistry, Hansraj College University Enclave, University of Delhi Delhi 110007 India brijeshrathi@hrc.du.ac.in; Department of Chemistry, Miranda House, University of Delhi Delhi 110007 India poonam.chemistry@mirandahouse.ac.in; Department of Chemistry, Massachusetts Institute of Technology Cambridge Massachusetts 02139 USA; Key Laboratory of Control of Quality and Safety for Aquatic Products, Ministry of Agriculture, Chinese Academy of Fishery Sciences Beijing 100141 China; Fire Chemistry Group, Centre for Fire, Explosives & Environment Safety Delhi-110054 India; South Ural State University, Laboratory of Computational Modeling of Drugs 454080 Russia potemkinva@susu.ru; Department of Chemistry, Aditi Mahavidyalaya, University of Delhi Bawana Delhi-110039 India

## Abstract

Low-molecular weight organic gelators have been of significant interest in recent years because of their interesting properties and potential applications in sensing technology, biomedicine and drug delivery. Herein, the synthesis, characterization and gelation properties of new oseltamivir conjugates are reported. The oseltamivir–triazole conjugate 1 was synthesized *via* a click-reaction in a 75% yield. The key features of this conjugate include the presence of amide, flexible ester linkages and a triazole scaffold linking a hydrophobic alkyl chain. The conjugate 1, possessing a long alkyl chain, showed gelation properties in various apolar organic solvents. This gelation behavior was not observed in the case of the deesterified conjugate 2; this indicated the necessity of the alkyl chain for gelation. The gelator 1 showed thermoreversible gelation properties in a range of linear alkane solvents (from *n*-pentane to *n*-dodecane). A scanning electron microscopic study suggests that the gelator 1 exists as cross-linked structures, which are self-aggregated in the range of submicrometers, as supported by extensive ^1^H-NMR studies. The rheological parameters supported the occurrence of a soft gelation process, and the gel formed in *n*-decane was found to be stiffer than that formed in *n*-hexane. Computational studies suggested that the gelation behavior was indeed due to micelle formation and dependent on the lipophilicity of solvents.

## Abstract 2

Low-molecular weight organic gelators have been of significant interest in recent years because of their interesting properties and potential applications in sensing technology, biomedicine and drug delivery.

## Graphical abstract

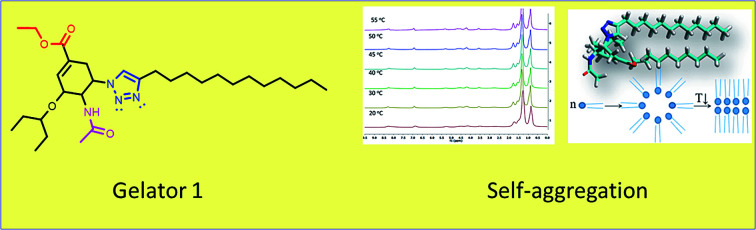

## Introduction

Low-molecular weight gelators (LMWG) are soft materials possessing the capability to construct three-dimensional networks stabilized by non-covalent interactions. Recently, a rapid growth in research on the use of small organic molecules as LMWG has emerged probably due to their interesting properties *i.e.* a distinct nanoscale one-dimensional molecular arrangement, flexibility associated with structural alterations, thermo-reversibility, sensitivity to the outside physical or chemical objects, small size of gelator molecules, and ease of synthesis of gelator molecules.^1–34^ Organogelators are under extensive investigation because of their potential applications such as in drug delivery,^1–4^ sensing technology,^5–28^ light-harvesting and optical devices,^26,28–34^ and oil spill recovery.^2,35–39^ The self-assembly of small molecules into a nanocomposite remains important in many areas of nanomaterials;^40^ the aggregation of nanoparticles to form a nanocomposite is a complicated process, and several methods have been implemented to overcome the related issues.^41,42^ The spontaneous self-association (gelation) of small molecules is directed by weak physical interactions such as hydrogen bonding (HB), van der Waals forces, π–π stacking, electrostatic interaction, dipole–dipole interaction, solvophobic interaction, and host–guest interaction.^43–48^ The gel strength is dependent on the nature of the chemical groups that facilitate non-covalent interactions in solvent media. Gelation could be a result of the assembly of small molecules into cross-linked structures maintained by the abovementioned weak forces in the presence of solvent molecules.^35,49–57^ Alternatively, the cross-linkages of an organogelator stabilized by additional supramolecular interactions between gelator molecular chains may be responsible for gelation, which is quite frequently observed for polymeric species. However, the assembly of low-molecular weight molecules into fibrous linkages involves very stable non-covalent interactions between individual small molecules to achieve an appropriate degree of polymerization to form a gel.

Although many organic gelators with diverse structures are available, there is a demand for new inexpensive, suitable and biocompatible small molecules exhibiting gelation properties in a variety of solvents. Small organic molecules possessing sufficient stability, chirality, H-bond donor or acceptor positions and low toxicity are considered as appropriate synthons for the development of low-molecular weight gelators. The organic gelators that can be synthesized following simple and convenient procedures and easily tailored are always preferred. In this context, a one-step and convenient click-reaction has been employed to generate several new low-molecular weight organic gelators.^58–64^ The interesting feature of this one-step reaction is that it results in the formation of new small molecules containing triazole that acts as a H-bond acceptor and a binding center for metal ions; for achieving new organogelators, we have selected 5-azido oseltamivir to implement the click-reactions on oseltamivir, anticipating the resulting conjugates as robust synthons for the development of new organic gelators with chirality, an amide linkage, a flexible ester bond and a triazole ring. Furthermore, the oseltamivir moiety provides high bioactivities and biocompatibility; however, its gelation properties have never been evaluated and reported in the literature. In this study, we report a convenient synthesis of new oseltamivir–triazole conjugates *via* a click-reaction and systematic studies of their gelation properties in several organic solvents. Several computational studies have been performed to investigate both the kinetic and the thermodynamic characteristics of gelation processes;^65–74^ however, majority of these processes are based on quantum methods or the methods of molecular or Brownian dynamics. Challenges such as laboriousness and long computational time impeded the study of datasets of molecules sufficient to generalize and determine the gelation conditions. Therefore, in the current study, we used a simple and alternative approach based on chemoinformatics that allowed us to suggest the mechanism of gelation and the factors that determine its possibility and identify the zones of gelation.

## Results and discussion

### Synthesis and characterization of oseltamivir–triazole conjugates

**Scheme 1:**
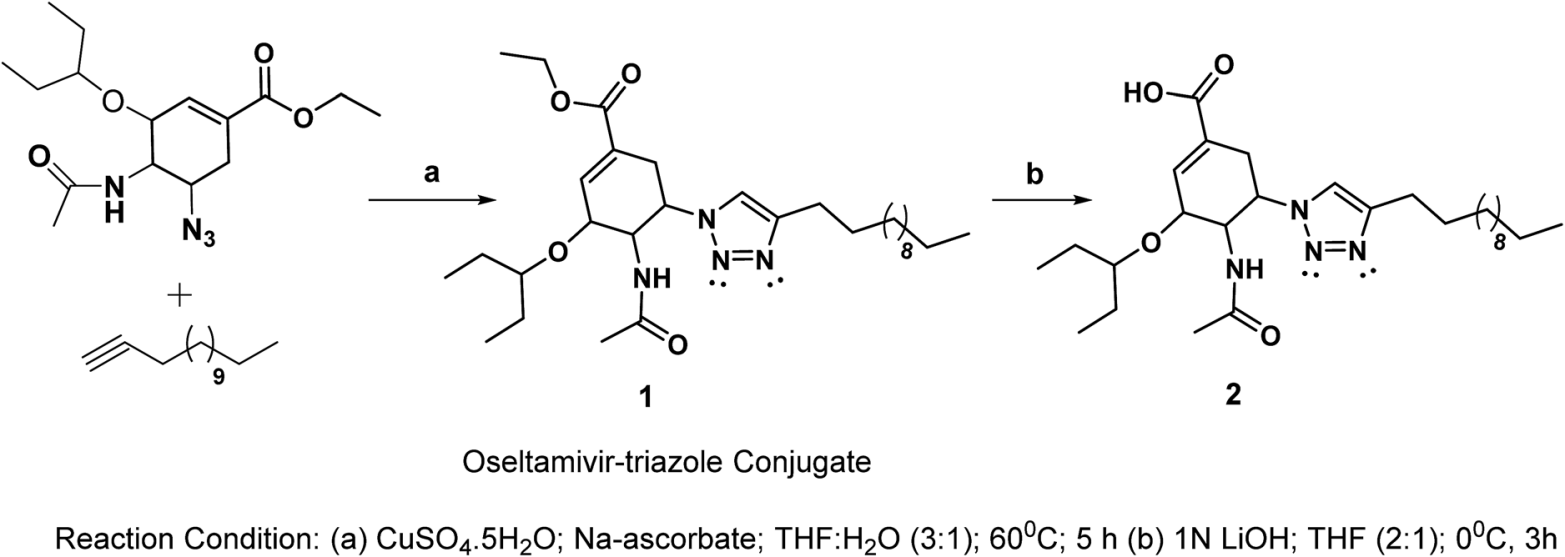
Synthesis of oseltamivir conjugates.

Considering the search for new low-molecular weight gelators (LMWGs), herein, we performed click-reactions to synthesize oseltamivir–triazole conjugates. The synthetic route for the designed oseltamivir-based gelators is shown in Scheme 1.

The key features of the new conjugate include the presence of amide, flexible ester linkages and a triazole scaffold linking a hydrophobic alkyl chain. The click reaction between 5-azido oseltamivir and 1-tetradecyne was accomplished in the presence of copper sulphate and sodium ascorbate to obtain the new conjugate 1 in a 75% yield. The conjugate 1 was deesterified in the presence of 1 N LiOH to afford 2 in a 74% yield. The compounds 1 and 2 were characterized by standard spectroscopic methods, from which satisfactory analytical data corresponding to their molecular structures were obtained (see the ESI†). An extensive NMR study (*i.e.* TOSY, COSY, HSQC and HMBC) was performed with the aim to deliver the appropriate interpretation of the characteristic peaks in the spectra. In the ^1^H NMR (CDCl_3_) spectrum of the conjugate 1, a doublet appeared at 7.05 ppm due to the proton of the triazole ring. The methylene and methyl protons of the ester moiety appeared at 4.08–4.22 (m) and 1.22–1.35 (m) ppm, respectively. The NH proton appeared as a broad singlet at 7.41 ppm. The methylene protons of the alkyl chain connected to the triazole ring were observed at 2.59 (t), which were slightly deshielded as compared to other methylene protons of the chain due to the anisotropic effect. The methylene protons associated with the long alkyl chain were observed in range of 1.22–1.35 ppm; however, terminal methyl protons were observed as a multiplet at 0.82–0.92 ppm.

### Gelation behavior

**Fig. 1:**
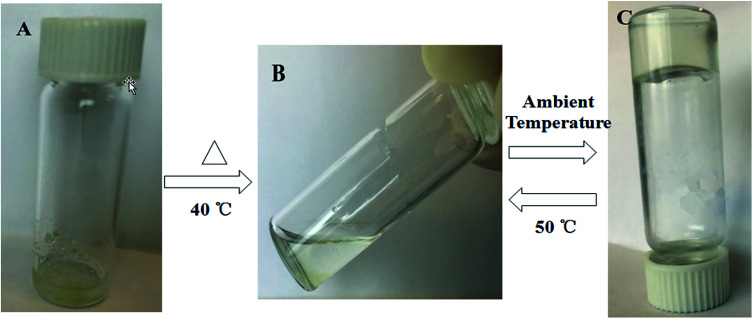
Schematic of the thermoreversible behavior of conjugate 1 in *n*-hexane: (A) an insoluble compound, (B) a solution, and (C) a transparent gel at ambient temperature.

The gelation capability of both conjugates 1-2 was examined in a wide range of solvents and mixture of solvents (Table 1). The conjugate 1 showed gelation ability in any linear alkane solvent such as *n*-hexane, *n*-heptane, *n*-octane, *n*-decane and *n*-dodecane; however, the deesterified conjugate 2 did not form a gel in any of the listed solvents. The conjugate 1 possesses a flexible ester linkage and is expected to form fibrous networks in apolar organic media as it acts as a hydrogen-bond accepter. The long alkyl chain and ester linkage could collectively be responsible for the gelation behavior of 1 mediated by linear alkane solvents. Transparent gelation was achieved in all the abovementioned solvents. Partial gelation behavior of the conjugate 1 was noticed in *n*-pentane and cyclohexane. This indicated that the alkyl chain present in the solvents was also crucial for the appropriate interactions between the solvent and the conjugate. Note that in other solvents, the gelation process was not observed. Instant and transparent gel formation was achieved for the conjugate 1 in *n*-hexane at ambient temperature. The conjugate 1 (5 mg) was added to *n*-hexane (300 μL) and warmed at 40 °C. The obtained homogeneous solution was then cooled down to room temperature to obtain the gel (Fig. 1). The excellent thermoreversibility of the gel was tested by heating the gel up to 50 °C that resulted in a clear solution, and the gelation could be brought back upon cooling this solution down to ambient temperature. This process was repeated more than three times to confirm the thermoreversible behavior of the gel. The gelation capabilities of 1 in linear *n*-alkane solvents could be associated with the hydrophobic interactions facilitated by the long triazole-alkyl chain and ester linkage. Remarkably, the presence of an ester linkage is crucial for gelation as conjugate 2, which does not contain an ester linkage, does not show gelation in any of the listed solvents (Table 1).

**Table 1:** Gelation properties of the conjugate 1 and 2 in various organic solvents [PG = partial gel; TG = transparent gel; I = insoluble; S = solution; and *T*_gs_ = gel–sol transition temperature]

Solvents	1	2	*T*_gs_	log *P*
*n*-Pentane	PG	I	30 °C	2.77
*n*-Hexane	TG	I	42 °C	3.26
*n*-Heptane	TG	I	45 °C	3.75
*n*-Octane	TG	I	48 °C	4.24
*n*-Decane	TG	I	52 °C	5.22
Dodecane	TG	I	55 °C	6.19
Cyclohexane	PG	I	—	2.71
Cyclohexanone	I	I	—	1.66
1-Propanol	S	S	—	0.31
2-Propanol	S	S	—	0.32
Benzene	S	S	—	2.02
Toluene	S	S	—	2.59
DMSO	S	S	—	0.79
DMF	S	S	—	−0.62
Diethyl ether	S	S	—	1.26
Chloroform	S	S	—	1.69
Dichloromethane	S	S	—	1.42
Carbon tetrachloride	S	S	—	2.45
THF	S	S	—	0.76
Water	I	I	—	−0.19
Methanol	S	S	—	−0.45
Ethanol	S	S	—	−0.10
DMSO : water (3 : 1)	S	—	—	0.55
DMF : water (3 : 1)	S	—	—	−0.51
MeOH : water (3 : 1)	S	—	—	−0.38
Hexane : ethyl acetate (1 : 10)	S	—	—	1.22
Hexane : chloroform (1 : 10)	S	—	—	1.83

Perhaps, the ethyl group of the ester linkage mediates the formation of three-dimensional networks *via* van der Waals interactions with the triazole-linked long alkyl chain. Note that gelation was noticed to be more rapid with an increase in the carbon chain length of the solvent. For instance, in hexane, the gelation was quite slow, whereas in decane and dodecane, gel formation was very fast. In the UV-visible spectra, we could notice a significant blue shift upon gelation in all the listed solvents (see the ESI†).

**Fig. 2:**
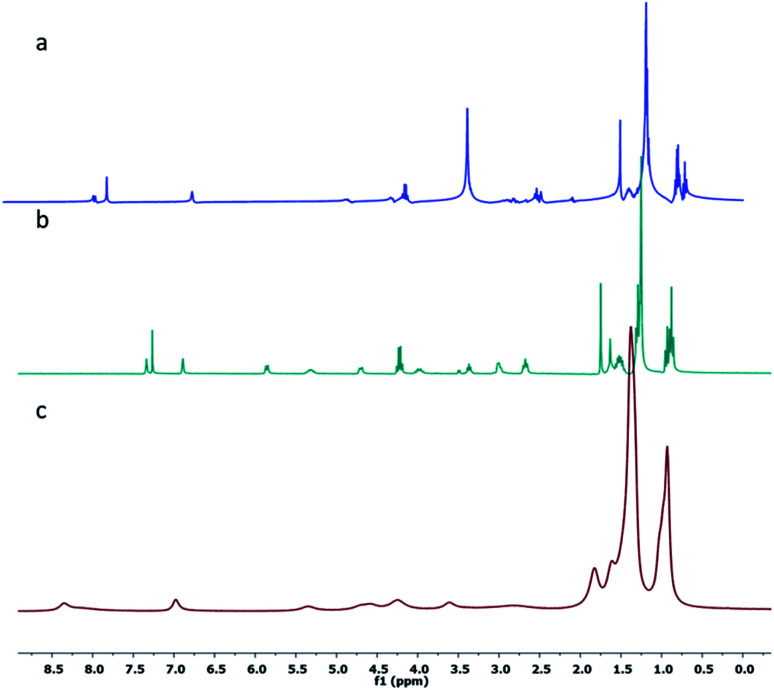
^1^H NMR spectra of the gelator 1 at ambient temperature in (a) DMSO-d_6_, (b) CDCl_3_ and (c) hexane-*d*_14_.

Furthermore, ^1^H-NMR studies were carried out in different solvents such as *n*-hexane-*d*_14_, CDCl_3_ and DMSO-*d*_6_. It was observed that the ^1^H-NMR spectra of 1 in CDCl_3_ and DMSO-*d*_6_ showed splitting of peaks due to coupling of neighboring protons, whereas in the ^1^H-NMR spectrum of 1 in *n*-hexane-*d*_14_, broadening of the peaks was observed. As shown in Fig. 2a and b, the terminal methyl protons at 0.87 ppm showed a multiplet in CDCl_3_ and DMSO-*d*_6_; however, a broad signal (with no splitting) was observed in *n*-hexane-*d*_14_ (Fig. 2c), indicating self-aggregation of 1 in *n*-hexane.

**Fig. 3:**
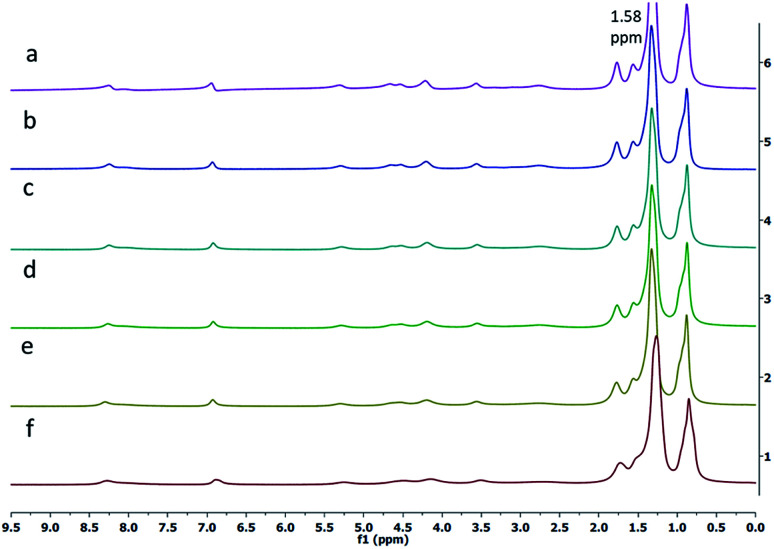
Variable temperature ^1^H NMR spectra of the gelator 1 in hexane-*d*_14_ at (a) 55 °C, (b) 50 °C, (c) 45 °C, (d) 40 °C, (e) 30 °C and (f) 20 °C.

Next, the ^1^H-NMR spectrum of the gelator 1 was obtained in *n*-hexane-*d*_14_ at various temperatures. It was observed that the intensity of the peak for the alkyl protons at 1.58 ppm increased as the temperature was increased from 20 °C to 55 °C. The broad signals for alkyl protons were observed at lower temperatures (20 °C, Fig. 3f), whereas at higher temperatures (55 °C, Fig. 3a), the peaks for alkyl protons appeared sharp, supporting the thermoreversible behavior of the gelator 1.

### Rheological measurements

We also studied the rheological parameters of the gels prepared in different solvents, for example, the gelation of 1 in *n*-hexane and *n*-decane. Measurement of the rheological parameters is critical to judge the gelation process.^75–78^ To investigate the effect of the solvents, the gels separately prepared in *n*-hexane and *n*-decane were considered for the rheological study (see the ESI†). In both solvents, the materials were observed as gels since *G*′ > *G*′′ for all the frequencies tested, and hence, we could say that the gelator in both solvents was crosslinked. The gels formed in *n*-decane were found to be stiffer than the gels formed in hexane since their elastic modulus (*G*′) was greater. The loss modulus (*G*′′) is more frequency-dependent for the decane gels than that of the hexane gels; this indicates that the *n*-decane gels can dissipate more energy at higher frequencies than the hexane gels. The shear viscosity data shows that both gels are shear thinning gels; however, the magnitudes of their viscosities are different.

### Morphological study

**Fig. 4:**
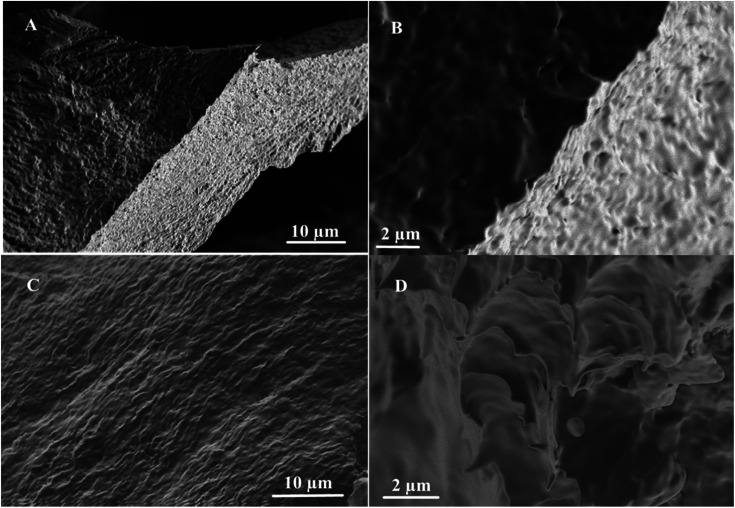
Schematic of the SEM images of 1 in (A and B) *n*-hexane (10 μm and 2 μm) and (C and D) *n*-heptane (10 μm and 2 μm).

To gain insights into the morphologies of the molecular assembly, the gelator 1 was examined by the SEM analysis, and the results are shown in Fig. 4. The SEM analysis was carried out in two apolar solvents: *n*-hexane and *n*-heptane. It has been observed from the analysis of the SEM images that the gelator 1 exists as cross-linked structures, which are self-aggregated in the range of submicrometers (2–10 μm). The morphological view of the gels formed in *n*-hexane shows spongy aggregates (Fig. 4A and B). On the other hand, the aggregation in *n*-heptane changes slightly and has been observed to assume a fiber-like structure (Fig. 4C and D).

We noticed a slight change in the morphological view of the gels in both solvents. For example, in *n*-hexane, the spongy cross-linked self-association of the conjugate 1 (Fig. 4A) slightly changes to interconnected dense fibrous structures (Fig. 4C). Furthermore, cloud-like structures were observed in *n*-heptane, as shown in Fig. 4D. These observed morphological differences could be attributed to the various assembly modes and forces between the gelator and the solvent molecules.

**Fig. 5:**
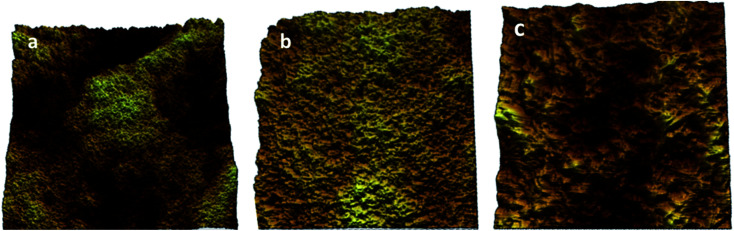
Schematic of the AFM images of 1 in *n*-hexane: (a) 50 μm, (b) 20 μm and (c) 7 μm.

Furthermore, the topographic data of the gelator 1 was examined using atomic force microscopy (AFM) analysis in the tapping mode to confirm the self-aggregation of 1 in *n*-hexane. The AFM analysis was performed in the submicrometer range of 7–50 μm, as shown in Fig. 5(a–c). The formation of small globular structures over the scanned area of the conjugate 1 supported the spongy cross-linked self-association of the gelator 1 in *n*-hexane, as observed *via* the SEM studies.

### Computational study

To clarify the causes of the gelation of conjugates in a series of solvents, a computational study was performed. Note that the formation of gels was observed for the solutions of conjugate 1 in saturated hydrocarbons, and transparent gels were formed in acyclic hydrocarbons with more than five carbon atoms. In solvents, *i.e.* pentane and cyclohexane, having shorter chain lengths (the latter has a shorter length due to its cyclic structure), partial gels are formed. In other solvents, a gelation process is not observed under the experimental conditions. For the conjugate 2, a gelation process was not observed in any of the abovementioned solvents.

**Fig. 6:**
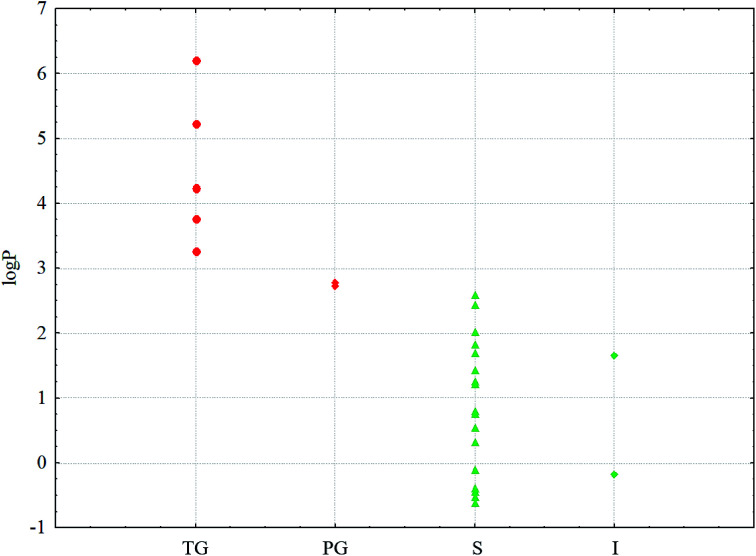
log *P* values for the solvents and phases: 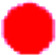 – TG; 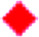 – PG; 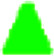 – S; 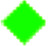 – I. Red are the phases that form gels; green are the phases that do not form gels.

A characteristic feature of higher saturated hydrocarbons is their high lipophilicity. Therefore, for all the solvents, log *P* values (*P* is the distribution coefficient in the *n*-octane–water system) as well as several physicochemical descriptors were calculated online on the web portal http://www.chemosophia.com.^79,80^ It has been shown that gel formation is indeed dependent on the lipophilicity of solvents. In solvents whose log *P* value is higher than 2.7 (see Fig. 6 and Table 1), gelation has been observed. It can be speculated that gelation involves hydrophobic and van der Waals interactions since these interactions are typical of aliphatic hydrocarbons. Indeed, log *P* in this series of compounds correlates well with the energy of van der Waals interactions (correlation coefficient *R* = 0.826). Moreover, the formation of transparent gels was observed for solvents with log *P* above 2.8. For solvents with log *P* below 2.7, either solutions or insoluble sediments were formed. Note both the conjugates have an amphiphilic structure with a polar head and a non-polar tail. These molecules can form micelles or inverse micelles in solutions depending on the solvent used. The former are usually formed in polar solvents, whereas the latter are usually formed in non-polar solvents. Most likely, micelles form in solutions, as evidenced by the blue shift in the UV spectrum, which is observed not due to absorption (the molecules do not contain typical chromophores), but by the scattering of blue light, which is common in colloidal systems. When the temperature decreases below *T*_gs_, the micelles can “open” and merge into bilayers with gel or even liquid crystal phases, which is typical, for example, of lipids.^81,82^ At lower temperatures, crystallization is possible. Often, the crystals possess a hexagonal order with bilayer motifs. In these experiments, the process of gelation with the formation of bilayers is typical of inverse micelles formed in non-polar aliphatic and alicyclic solvents.

**Fig. 7:**
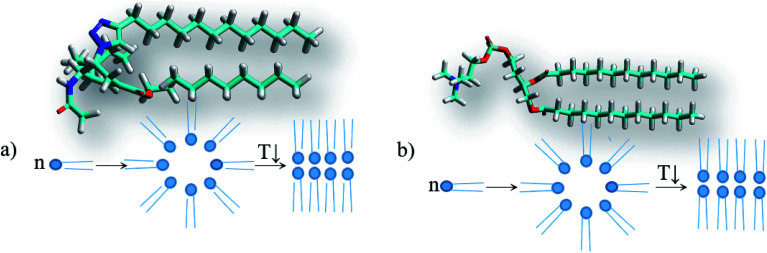
Structures and schematic of the particles, micelles and bilayers for (a) the complex of conjugate 1 with alkanes (using octane as an example) and (b) 1,2-dilauroyl-*glycero*-3-phosphatidylcholine.

However, the solvents in which gels are not formed also include non-polar and low-polar solvents such as the aromatic hydrocarbons benzene and toluene, carbon tetrachloride and diethyl ether. Therefore, to verify the abovementioned hypothesis, the solvation complexes of conjugates with solvent molecules were modeled using the MOPS algorithm.^83–88^ It is shown that the molecules of aliphatic and alicyclic solvents interact with the tails of the conjugates due to van der Waals interactions and form complexes with the structure shown in Fig. 7a. This structure is very similar to the structure of lipids (Fig. 7b). Similar to typical lipids, the complex has a polar head containing potential centers of hydrogen bonds (acetamide group) and two non-polar tails where the solvent molecule plays the role of the second tail. This complex can behave like a typical lipid, *i.e.* it can form inverse micelles in solutions at high temperatures and gel or liquid crystals with bilayer motifs at lower temperatures. It is possible that this mechanism for the inclusion of a solvent in a micelle can be realized for other surfactants. The deesterification of the conjugate 1 leading to the conjugate 2 weakens the intermolecular interactions of the solvent with the conjugate by an average of 14.4 ± 2.3 kJ mol^−1^ due to the disappearance of the interaction with the ethoxy group. Moreover, deesterification enhances the polarity of the head and adds potential centers of hydrogen bonds (carboxyl group), which yields an increase of head-to-head interactions and the formation of insoluble crystals with bilayer motifs under the experimental conditions, analogous to the case of lipids. The energy calculations were carried out using the MERA model.^79,80^ When the temperature is increased to the melting temperature, the crystals should melt with the possible formation of a gel or liquid crystal phase.

**Fig. 8:**
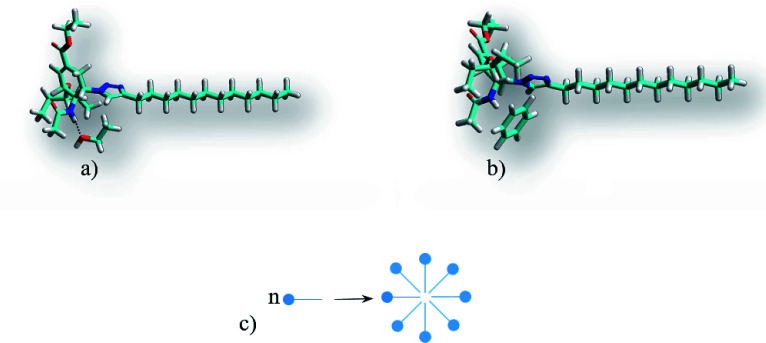
Structures of the conjugate 1 complexes: (a) with polar solvents (using ethanol as an example) and (b) aromatic solvents (using benzene as an example) and (c) the micelle formation scheme.

**Fig. 9:**
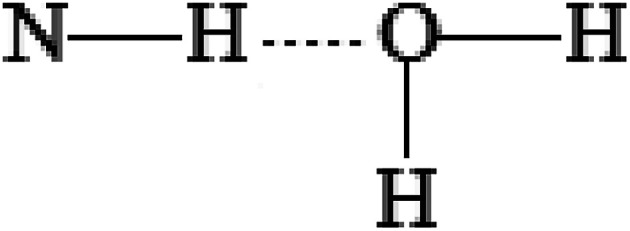
A hydrogen-bonded complex formed by a conjugate and water.

Simulation of the solvate complexes for conjugates with oxygen- and chlorine-containing solvents shows that these solvents prefer to form hydrogen bonds or Cl⋯H contacts of oxygen or chlorine of the solvent with amide hydrogen of the conjugate, as shown in Fig. 8a, which block and change the conformation of the polar acetamide head and do not allow the formation of inverse micelles, as in the case of complexes of saturated hydrocarbons with the conjugate 1. Moreover, in polar solvents, the formation of micelles (not inverse micelles), as shown in Fig. 8c, is more typical, such that the polar heads are in the polar solvent environment. Low-polar diethyl ether and non-polar carbon tetrachloride also form a hydrogen bond and Cl⋯H contact, respectively, with the amide hydrogen of conjugates. Aromatic solvents (benzene and toluene) also interact with the amide hydrogen of the conjugates, leading to its interaction with the π-electron system of the aromatic ring, as shown in Fig. 8b. Structures of the solvate complexes for oxygen- and chlorine-containing solvents differ slightly for the conjugates 1 and 2; therefore, in most cases, micellar solutions are formed at room temperature. The exception is water, in which insoluble sediments are formed for both conjugates. This happens due to the presence of two hydrogen atoms that are able to form hydrogen bonds and the absence of steric obstacles for it. Therefore, water not only maintains the potential hydrogen bond center of the head of the conjugates, but also enhances it. In the hydrogen-bonded complex formed by a conjugate and water (as shown in Fig. 9), both hydrogens of water form hydrogen bonds with the carbonyl groups of neighboring conjugates; this leads to an increase in head-to-head interactions and the formation of insoluble sediments under experimental conditions.

**Fig. 10:**
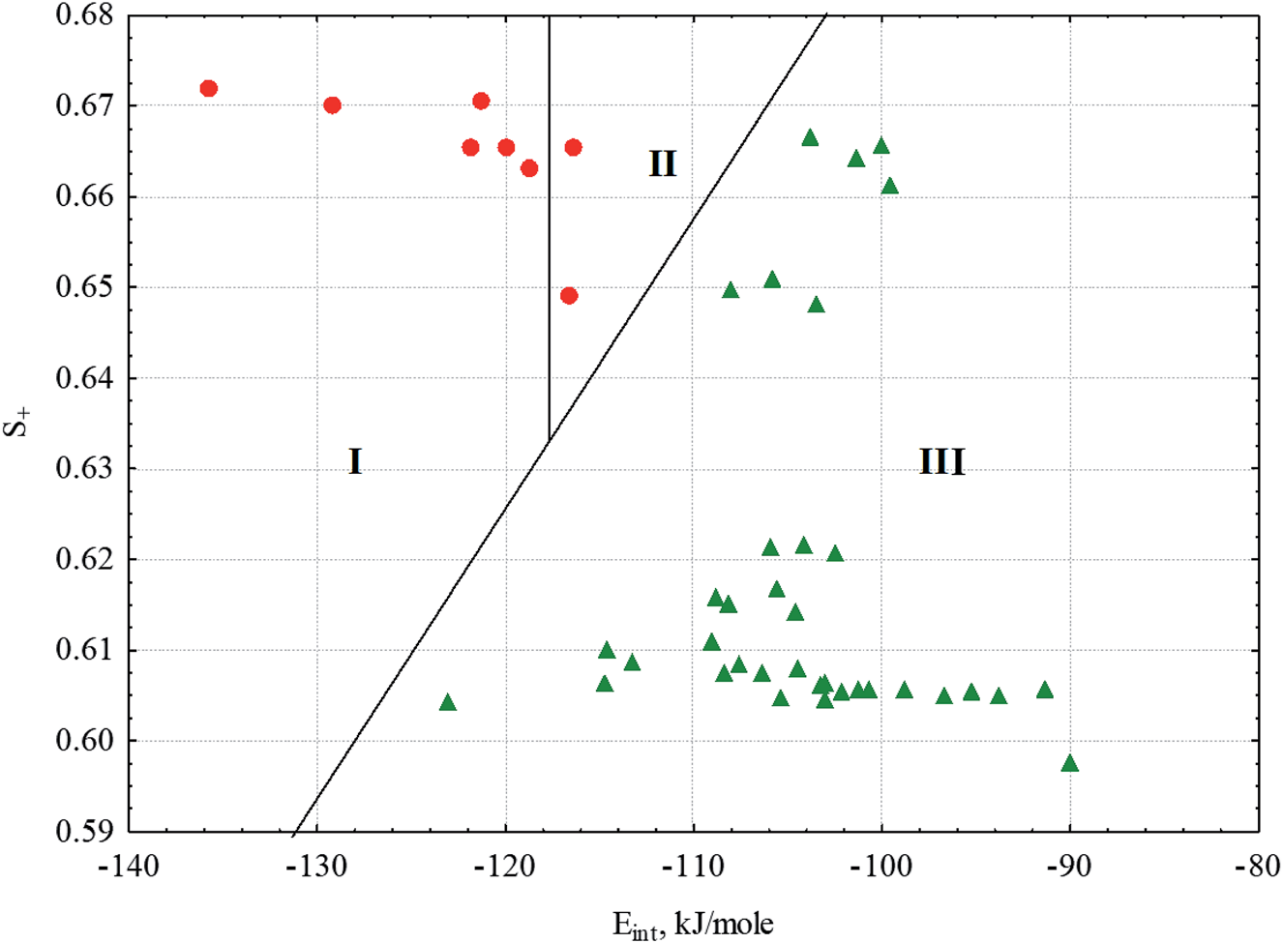
Complexes of the conjugates 1 and 2 with solvents in the factor space of the energy of intermolecular interactions (*E*_int_) and the portion of positively charged area (*S*_+_). 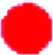 – complexes forming gels; 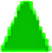 – complexes not forming gels. I – zone of formation of transparent gels; II – zone of formation of partial gels; and III – zone of complexes not forming gels.

If we consider all the complexes of the conjugates 1 and 2, the distinctive quantitative feature of the complexes forming gels is the lower energy of intermolecular interactions in the solvation complexes (*E*_int_), which varies in the range of −122.5 ± 6.7 kJ mol^−1^. For all other complexes of the conjugates 1 and 2, *E*_int_ varies in the range −104.1 ± 6.5 kJ mol^−1^. Energy calculations were carried out within the MERA model.^79,80^ The only exception is the complex of conjugate 2 with dodecane, for which *E*_int_ = −123.1 kJ mol^−1^ falls within the confidence interval of the substances forming gels. Another quantitative characteristic distinguishing the solvate complexes forming gels from those not forming gels is the portion (dole) of the positively charged surface of the complexes (*S*_+_), also calculated within the MERA model.^79,80^ For the complexes forming gels, *S*_+_ is higher due to the presence of ethyl group attached to ester linkage in conjugate 1. The value of *S*_+_ varies within 0.6651 ± 0.0072. For complexes that do not form gels, *S*_+_ varies within 0.619 ± 0.021. In the space of these two factors, the classes of complexes forming and not forming gels are well distinguished (Fig. 10); this makes it possible to predict gelation in other solvents or with other amphiphiles. In Fig. 10, we can well distinguish the zone of transparent gel formation, the zone of partial gel formation (in this zone, only 2 complexes of conjugate 1, with pentane and cyclohexane, are formed) and the zone of complexes that do not form gels. Thus, having calculated *E*_int_ and *S*_+_ for a new system, we can speculate its phase state.

Among the solvate complexes that do not form gels, there are 2 classes of complexes. These are solvate complexes forming solutions at room temperature and those forming insoluble sediments. Note that these classes differ well using solvent characteristics: solutions are formed only in solvents ensuring the formation of micelles (Fig. 8c), except for water (the reasons for this have been discussed above); other solvents (saturated hydrocarbons with conjugate 2) probably form crystals with bilayer motifs, as shown in Fig. 8a. Cyclohexane deviates from this rule.

The better distinguishing quantitative feature of the systems that form solutions at room temperature and those that form insoluble sediments is the total energy of solvents calculated using the MERA model.^79,80^ For solvents in which the solutions are formed, the total energy varies in the range −32 ± 14 kJ mol^−1^, whereas for solvents in which sediments are formed, the total energy varies in the range +17 ± 25 kJ mol^−1^. Thus, more energetically beneficial solvents (having a network of hydrogen bonds, strong intermolecular Coulomb or π-stacking interactions, *etc.*) support the formation of micelles (Fig. 8c). Cyclohexanone is no exception from this rule. Apparently, its intermolecular interaction energy is insufficient for micelle stability. The only exception is water (the reasons for this have been discussed above).

### Conclusion

In summary, herein, new oseltamivir–triazole conjugates were developed as low-molecular weight thermoreversible gelators. A simple and convenient click-reaction was employed to prepare the new conjugates. The gelation behavior was noticed exclusively in linear alkyl solvents; this indicated that hydrophobic interactions mediated this process. The self-association of the conjugate was also supported by extensive ^1^H-NMR and computational studies.

We observed that the new conjugate 1 acted as a thermoresponsive organic gelator in linear alkane solvents as supported by the variable-temperature NMR studies. We studied the effect of solvent alkyl chain length on the gelation ability. The elasticity properties and morphologies of the gelator were studied with respect to gelation in different solvents. Furthermore, the conjugate 1 was treated with LiOH to afford the de-esterified conjugate 2 that did not show gelation ability in any of the listed solvents. This indicated the importance of the ethyl ester linkage for the construction of three-dimensional networks. The causes of gelation in many amphiphile–solvent systems have been elucidated with the aid of computational approaches. The solvate complexes in these systems were modeled, and the structures of the aggregates in different phase states were suggested. Overall, we attempted to investigate oseltamivir conjugates as a new entry to low-molecular weight thermoreversible gelators in a range of linear alkane solvents.

## Experimental

### General

All reagents and solvents purchased from commercial sources were used without further purification. The starting material 5-azidooseltamivir was purchased from Carbosynth Limited (Berkshire, UK). Reactions were monitored by thin-layer chromatography (TLC) EMD TLC silica gel 60, which were analysed using UV-light. Nuclear magnetic resonance (NMR) spectra were obtained using the VARIAN Mercury 300 NMR Spectrometer (Oxford Instruments Ltd.) operating at ambient probe temperature (293 K) in the MIT Department of Chemistry Instrumentation Facility (DCIF). ^1^H-NMR spectroscopy in DMSO-*d*_6_ was carried out using JEOL ECX-400P NMR at 400 MHz at USIC, University of Delhi. Chemical shifts were reported in ppm with tetramethylsilane (TMS) as an internal standard. The VT-NMR experiments were carried out using the VARIAN Inova-500 NMR Spectrometer obtained from Oxford Instruments Ltd. in the MIT Department of Chemistry Instrumentation Facility (DCIF). ESI-MS data were collected on a LC-MS (ESI) apparatus using the Agilent 1260 LC system (column: Agilent Poroshell 120, EC-C18, 2.7 μm pore size) and the Agilent 6230 TOF system containing the Agilent Jetstream ESI source. LC-MS grade acetonitrile and water containing 0.1% formic acid were used for the solvent system. The morphologies of the gels were characterized using scanning electron microscopy (SEM, JEOL 5910). The gels for SEM analysis were prepared by vacuum freeze-drying of the gel formed in the solvent for 12–24 h. The dried samples held on glass substrates were attached to a copper holder for SEM using a conductive adhesive tape. The topographic surfaces of gels were characterized using atomic force microscopy (AFM, Flex-Axiom C3000). X-ray powder diffraction was carried out on a PaNalytical XPert Pro MRD HR XRD system. The rheological parameters were studied on a stress-controlled TA Instruments AR-G2 rheometer using a 40 mm 2 degree cone and plate fixture.

### Synthesis and characterization

A round-bottom flask was charged with 5-azidooseltamivir (1.50 mmol), THF : H_2_O (2 : 1; v/v; 20 mL) and 1-tetradecyne (1.60 mmol) under inert conditions. To the reaction flask, CuSO_4_·H_2_O and Na-ascorbate (each 20 mol%) were added, and the contents were stirred at 40 °C for 5 h. After completion of the reaction, as monitored by TLC, the reaction contents were cooled down to ambient temperature and concentrated under reduced pressure. The obtained residue was extracted with ethyl acetate, and the organic layer was washed with excess distilled water to remove inorganic impurities from the product. The organic layer was dried over anhydrous magnesium sulphate and concentrated under *vacuo*. Thus, the obtained product was purified by silica gel column chromatography eluted with chloroform, and the final compound 1 was isolated.

#### Ethyl 4-acetamido-5-(4-dodecyl-1*H*-1,2,3-triazol-1-yl)-3-(pentan-3-yloxy)cyclohex-1-ene-1-carboxylate (1)

Off white powder; MF: C_30_H_52_N_4_O_4_; yield 75%; *R*_F_ value: 0.50 (ethyl acetate : hexane; 1 : 1); ^1^H NMR (300 MHz, CDCl_3_): *δ* 7.41 (s, 1H), 7.05 (d, 1H), 6.87 (s, 1H), 5.25 (m, 1H), 4.60 (d, 1H), 4.08–4.22 (m, 3H), 3.36–3.40 (m, 1H), 2.84–3.04 (m, 2H), 2.59 (t, 2H), 1.49–1.58 (m, 6H), 1.22–1.35 (m, 22H), 0.82–0.92 (m, 10H); ^13^C NMR (75.5 MHz, CDCl_3_): 171.23, 165.92, 139.18, 128.38, 82.80, 74.90, 61.34, 57.68, 56.30, 32.33, 32.18, 30.05, 29.94, 29.90, 29.86, 29.69, 29.62, 29.51, 26.50, 25.93, 25.86, 22.95, 14.46, 14.39, 9.89, 9.51.

The de-esterification of compound 1 (0.094 mmol) was achieved in the presence of 1 N LiOH (1 mL) and THF : H_2_O (2 : 1; v/v; 3 mL) at 0 °C. Initially, the reaction contents were stirred at 0 °C for ∼1 h and then at ambient temperature for 3 h. The progress of the reaction was confirmed by TLC and after completion, the reaction mixture was diluted with ethyl acetate and 1 N HCl (∼5–6 pH) under cold conditions. The organic layer was separated and washed with distilled water and dried over anhydrous magnesium sulphate. The organic layer was concentrated, resulting in title compound 2 as a white solid.

#### 4-Acetamido-5-(4-dodecyl-1*H*-1,2,3-triazol-1-yl)-3-(pentan-3-yloxy)cyclohex-1-ene-1-carboxylic acid (2)

White powder; MF: C_28_H_47_N_4_O_4_; yield 74%; ^1^H NMR (75.5 MHz, CDCl_3_): *δ* 7.50 (s, 1H), 6.96 (s, 2H), 5.29 (s, 1H), 4.61 (s, 1H), 4.18 (s, 1H), 3.37 (t, 1H), 2.91–3.06 (m, 2H), 2.65 (t, 2H), 1.74 (s, 3H), 1.44–1.62 (m, 6H), 1.23–1.31 (m, 18H), 0.83–0.92 (m, 9H). MS (ESI-LCMS) *m*/*z* (%): 505.5440 (100) [M + H]^+^, 527.5303 (60) [M + Na]^+^.

### Gelation test

A fixed amount of compound (5 mg) was placed in a glass sample vial and mixed with the solvent (300 μL); the contents were heated until all solid substances were dissolved and then were stirred by vortex for 5 minutes. Thereafter, the obtained solution was cooled to room temperature and the vial was shaken well and inverted to examine gel formation.

### Computational details

Geometry optimization and calculation of geometry, charge, energy and physicochemical characteristics (*e.g.*, logarithm of octanol–water partition coefficient log *P*) of all individual molecules and complexes were carried out online using the ChemoSophia software and the original MOPS algorithm^73–78^ with continual account of solvents.

## Conflicts of interest

We declare that we have no conflict of interest.

## Supplementary Material

RA-009-C9RA02463H-s001
