# Supplementary material for: Theoretical and experimental studies of an oseltamivir–triazole-based thermoresponsive organogel
Source: RSC Adv. 2019 Jul 4;9(36):21031–41. doi: 10.1039/c9ra02463h (PMC9065751; doi:10.1039/c9ra02463h)
Supplement: RA-009-C9RA02463H-s001 [file RA-009-C9RA02463H-s001.pdf]

Electronic Supplementary Material (ESI) for RSC Advances.
This journal is © The Royal Society of Chemistry 2019

# Theoretical and Experimental Studies of Oseltamivir-triazole Based Thermoresponsive Organogel

Sumit Kumar[1,2,†], Lidong Wu[3,4,†], Neha Sharma[1,†], Ayushee[1], Kumar Kaushik[5], Maria Grishina[6], Bhupendra S Chhikara[7], Poonam[2,*], Vladimir Potemkin[6,*], Brijesh Rathi[1,*]

[1]*Laboratory for Translational Chemistry and Drug Discovery, Department of Chemistry, Hansraj College University Enclave, University of Delhi, Delhi 110007 India*
[2]*Department of Chemistry, Miranda House, University of Delhi, Delhi 110007 India*
[3]*Department of Chemistry, Massachusetts Institute of Technology, Cambridge, Massachusetts 02139, United States*
[4]*Key Laboratory of Control of Quality and Safety for Aquatic Products, Ministry of Agriculture, Chinese Academy of Fishery Sciences, Beijing 100141, China*
[5]*Fire Chemistry Group, Centre for Fire, Explosives & Environment Safety, Delhi - 110054*

[6]*South Ural State University, Laboratory of Computational Modeling of Drugs, 454080, Russia*
[7]*Department of Chemistry, Aditi Mahavidyalaya, University of Delhi, Bawana, Delhi-110039 India*

[†]These authors have contributed equally

**Corresponding Author**

Poonam (poonam.chemistry@mirandahouse.ac.in); Vladimir Potemkin (potemkinva@susu.ru); Brijesh Rathi (brijeshrathi@hrc.du.ac.in)

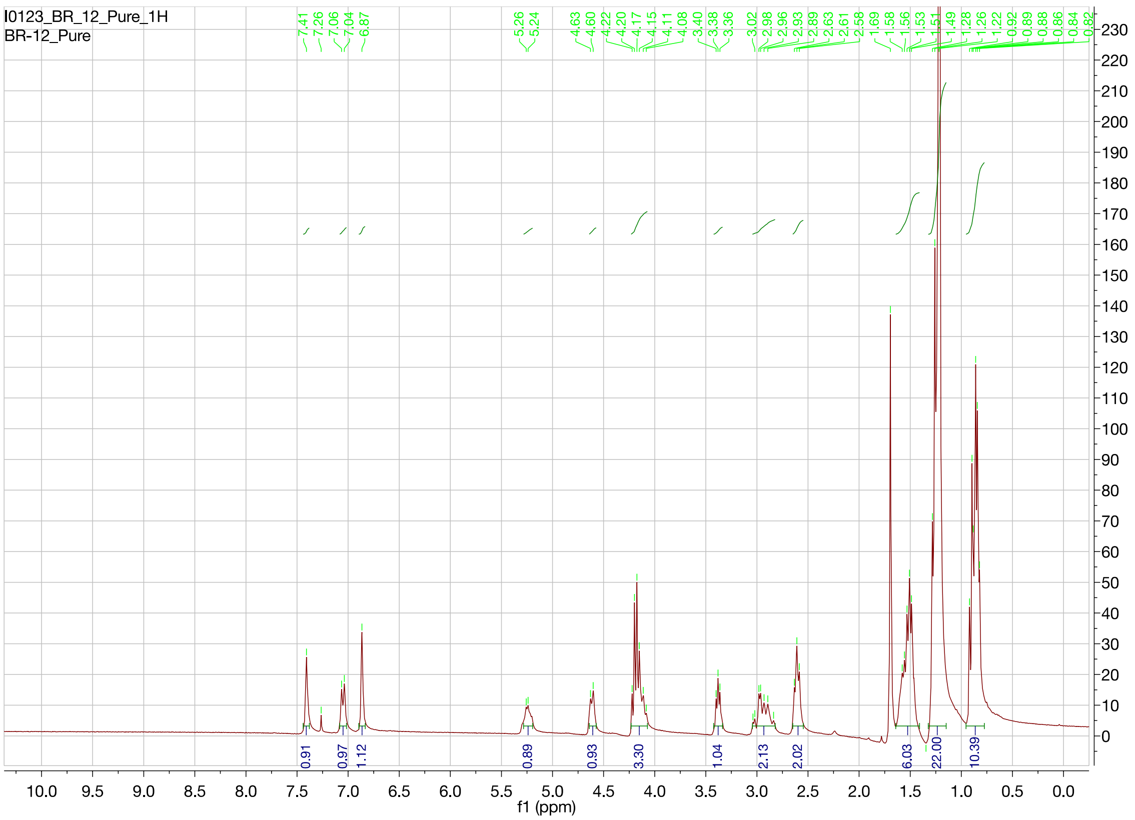

Fig. S1. $^{1}H$ NMR (300 MHz)

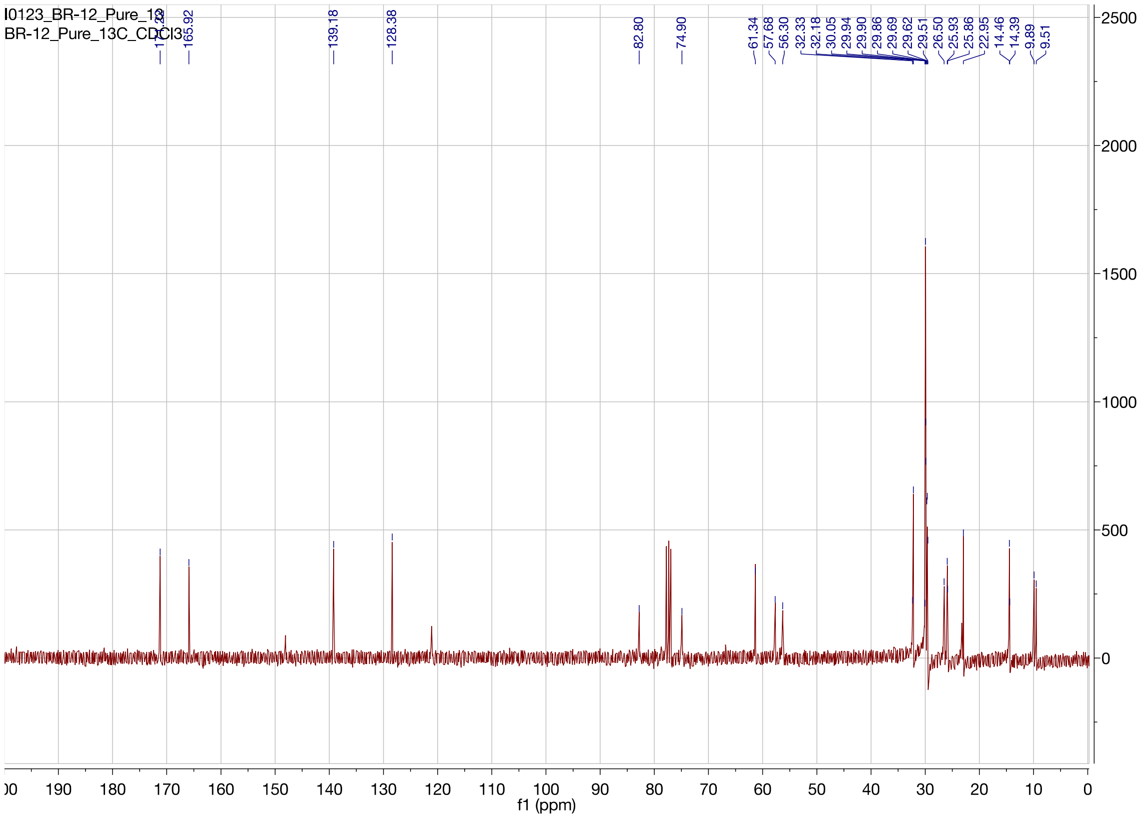

Fig. S2. $^{13}C$ NMR for Compound **1.**

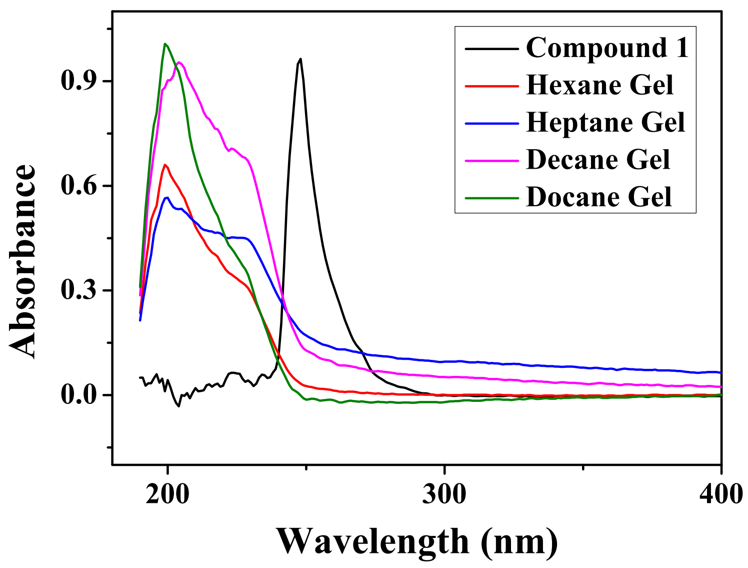


**Fig. S3.** UV-Visible Spectra for compound **1**.

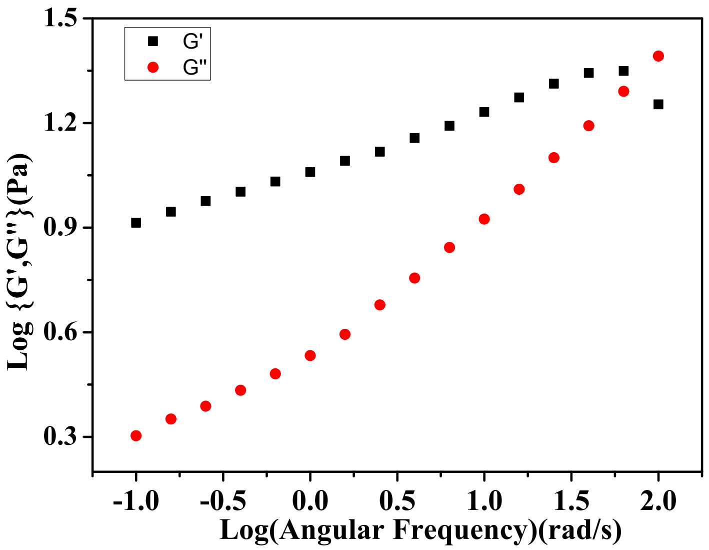


**Fig. S4**. Hexane frequency dependence studies of gelator **1**

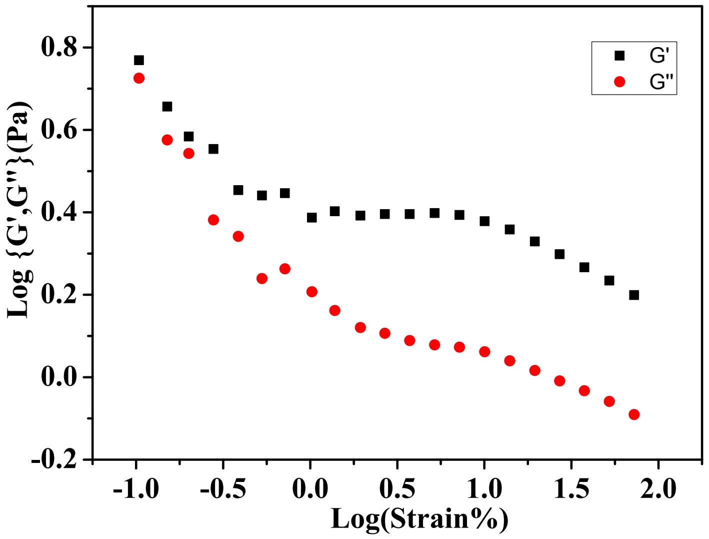


**Fig. S5**. Decane frequency dependence studies of gelator **1**

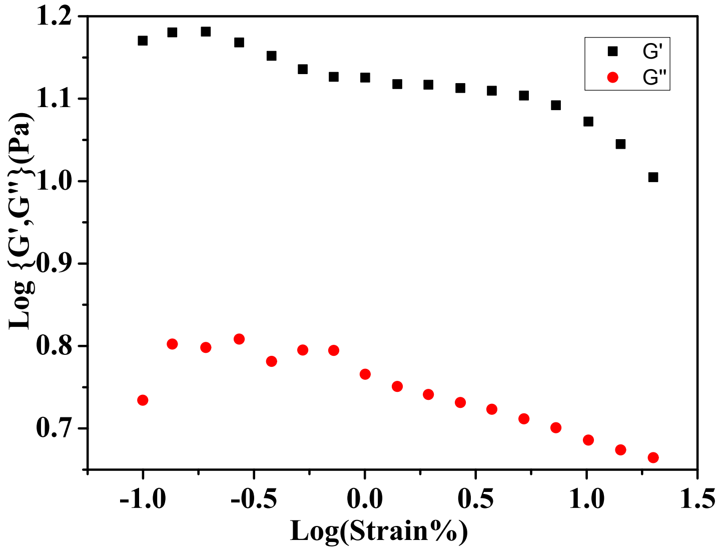


**Fig. S6.** Hexane strain and storage modulus for gelator **1**.

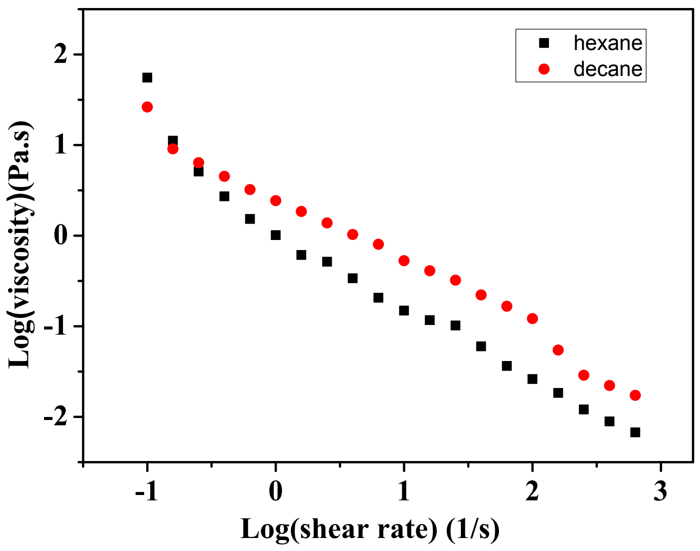


**Fig. S7.** Decane strain and storage modulus for gelator **1**.

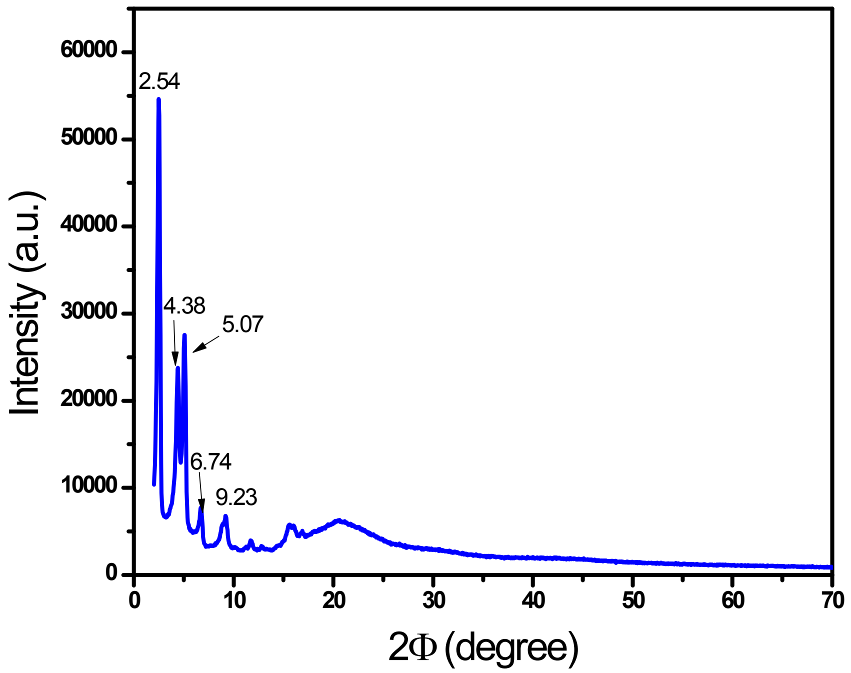


**Fig. S8.** Powder XRD of gelator **1**.
